# Review on Laser Technology in Intravascular Imaging and Treatment

**DOI:** 10.14336/AD.2021.0711

**Published:** 2022-02-01

**Authors:** Jing Li, Ce Shang, Yao Rong, Jingxuan Sun, Yuan Cheng, Boqu He, Zihao Wang, Ming Li, Jianguo Ma, Bo Fu, Xunming Ji

**Affiliations:** ^1^BUAA-CCMU Advanced Innovation Center for Big Data-Based Precision Medicine, School of Engineering Medicine, Beihang University, Beijing, China.; ^2^School of Biological Science and Medical Engineering, Beihang University, Beijing, China.; ^3^School of Instrumentation and Optoelectronic Engineering, Beihang University, Beijing, China.; ^4^Medical Engineering Devices of Xuanwu Hospital, Capital Medical University, Beijing, China.; ^5^China-America Institute of Neuroscience, Xuanwu Hospital, Capital Medical University, Beijing, China.; ^6^Key Laboratory of Big Data-Based Precision Medicine Ministry of Industry and Information Technology, Interdisciplinary Innovation Institute of Medicine and Engineering, Beihang University, Beijing, China.; ^7^Neurosurgery Department of Xuanwu Hospital, Capital Medical University, Beijing, China.

**Keywords:** laser technology, intravascular imaging, intravascular treatment, laser catheter, optical fiber

## Abstract

Blood vessels are one of the most essential organs, which nourish all tissues in our body. Once there are intravascular plaques or vascular occlusion, other organs and circulatory systems will not work properly. Therefore, it is necessary to detect abnormal blood vessels by intravascular imaging technologies for subsequent vascular treatment. The emergence of lasers and fiber optics promotes the development of intravascular imaging and treatment. Laser imaging techniques can obtain deep vascular images owing to light scattering and absorption properties. Moreover, photothermal and photomechanical effects of laser make it possible to treat vascular diseases accurately. In this review, we present the research progress and applications of laser techniques in intravascular imaging and treatment. Firstly, we introduce intravascular optical coherent tomography and intravascular photoacoustic imaging, which can obtain various information of plaques. Multimodal intravascular imaging techniques provide more information about intravascular plaques, which have an essential influence on intravascular imaging. Secondly, two laser techniques including laser angioplasty and endovenous laser ablation are discussed for the treatment of arterial and venous diseases, respectively. Finally, the outlook of laser techniques in blood vessels, as well as the integration of laser imaging and treatment are prospected in the section of discussions.

## 1.Introduction

Blood vessels constitute the first organ and form the largest network in our body, which supplies nutrients and oxygen, as well as removing metabolic waste [1, 2]. However, all the non-modifiable biological changes that occur within our blood vessels over time lead to a series of vascular diseases. Coronary artery disease (CAD) remains the leading cause of worldwide morbidity and mortality, specifically due to atherosclerosis and thrombosis [3]. In addition to coronary and intracranial vessels, peripheral arterial diseases (PAD) include diseases that cause obstruction of arterial blood flow, typically chronic arterial occlusive disease of lower limb [4]. Arterial thrombosis is an important cause of PAD and CAD, which leads to serious consequences such as acute myocardial infarction (AMI) and cerebrovascular diseases [5-7]. Vein disease generally refers to varicose veins or vein thrombosis, which is caused by slow blood flow and damage of the vessel walls [8, 9]. Deep vein thrombosis could cause pulmonary embolism and amputation [10, 11]. Therefore, intravascular imaging and treatment are indispensable for vascular diseases.

Existing clinical imaging techniques include ultrasound, computed tomography, magnetic resonance imaging, positron emission tomography/computed tomography, and single-photon emission computed tomography [12-14]. Conventional imaging techniques for vascular lesions provide anatomical and morphological information about vascular diseases, which cannot obtain intravascular images [15]. Intravascular imaging techniques, such as intravascular ultrasound (IVUS), optical coherence tomography (OCT), photoacoustic imaging (PAI), can obtain lumen size and length of vessel without the artifacts inherent to angiography [16]. Moreover, intravascular imaging provides high-contrast images of the lumen and stent, as well as visualization of the vessel wall in high resolution [17, 18]. IVUS overcomes the inherent limitations of other techniques, providing images of arterial walls and permitting detections of early-stage atherosclerosis as well as measurement of plaques [19]. However, IVUS lacks the resolution and specificity for detecting soft tissue type [20]. Compared with IVUS, image acquisition by OCT is faster with higher resolution, which offers more specific determination of tissue characteristics [21, 22]. However, OCT has a lower depth of tissue penetration [23]. PAI provides extremely high sensitivity regarding lipid distribution in atherosclerotic plaques [24, 25]. We introduce the state of the art of PAI and OCT, where laser technology is both utilized. In order to overcome the limitations of different imaging methods, multimodal imaging concepts have been considered in recent years [26]. Moreover, we summarize PAI and OCT by contrasting the strengths and limitations of each modality, as well as highlighting the potential applications of a multimodal system.

For vascular therapy, percutaneous coronary intervention (PCI) is commonly used in clinic, but PCI still has unexplained complications [27, 28]. In PCI, the use of balloon angioplasty is limited by the occlusion vessels and the restenosis vessels, which promotes the development of stents to maintain lumen integrity. The emergence of drug-eluting stents (DES) significantly reduces the incidence of restenosis and repeat revascularization after PCI [29]. However, there is still a risk of late thrombosis in current clinical outcomes [30]. Laser angioplasty, as a safe and high-precision method, can treat different CAD and PAD [31-33]. For intravenous therapy, some minimally invasive techniques have been introduced to reduce serious side effects, costs, and postoperative pain, such as ultrasound-guided foam sclerotherapy, radiofrequency ablation, and endovenous laser therapy (EVLA) [34]. Furthermore, EVLA is proved to be a safe and effective treatment that reduces pain, morbidity, recovery time, and recurrence rates, as well as improving the overall quality of life [35]. More importantly, imaging-guided laser technology for the treatment of intravascular diseases has an excellent prospect of clinical transformation. Therefore, we introduce the application and development of laser therapy in arterial and venous diseases.

**Figure 1.**
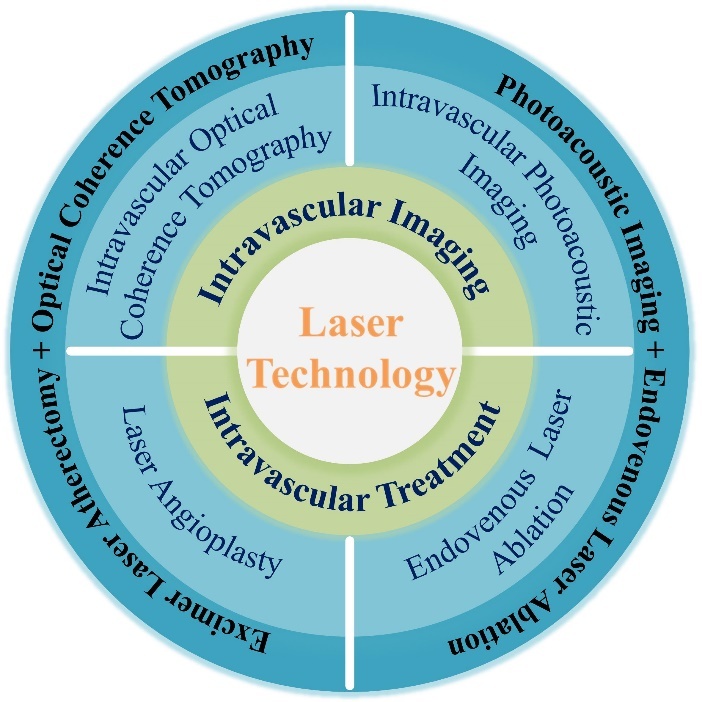
Laser technology used in intravascular imaging and therapy.

## 2.Research progress and applications of laser technology in intravascular imaging and treatment

At present, laser wavelengths range from ultraviolet to infrared bands. Laser devices tend to become compact and miniaturized, such as fiber lasers, laser diodes, and so on [36-38]. Meanwhile, fiber optic technique allows lasers to deliver energy through catheters into blood vessels. Laser technology is widely used in intravascular imaging and therapy (Fig. 1). Herein, we summarize the state of the art of laser wavelengths and fiber optic catheters in intravascular imaging and therapy. In addition, we compare the application parameters of intravascular laser imaging and therapy.

### 2.1*Laser technologies for intravascular imaging*

Intravascular laser technologies have provided new options to evaluate the various factors associated with atherosclerosis, plaque stability, and treatment of CAD [39]. One or more of these processes occur when light interacts with the tissue including the reflectance, refraction, scattering, absorption, and re-emission of the incident light [40]. The tissues in blood vessels have different absorption and scattering characteristics for different laser wavelengths. Therefore, laser can provide a light source for the imaging of specific tissue. Intravascular laser imaging techniques include OCT and PAI. OCT, also known as “optical biopsy”, produces high-resolution, micrometer-scale, and cross-sectional images of tissue structure by detecting echo delay time of back-scattered or back-reflected light [21, 22, 41]. The operational mode of OCT is akin to that of ultrasound imaging, whereas OCT uses light instead of sound. OCT systems based on Michelson interferometer rely on interference between a split and later recombined broadband optical field [42]. The axial and depth resolutions in OCT depend on the bandwidth of low-coherence light [43]. PAI is a novel optical imaging modality, which shows great promise in biomedical applications. The imaging modality is based on the photoacoustic effect discovered by Bell in 1880 [44]. The hybrid imaging modality merges optical absorption contrast and ultrasound image formation. The essential elements of PAI involve optical irradiation and absorption, ultrasonic detection, and image formation. The coherence and wavelength tunability of laser provide the advantages of imaging. Fiber optic technology is used to transmit light to the deep human tissues due to its flexibility and compactness [45]. In OCT and PAI, the combination of lasers and fiber optics is widely studied and applied, especially in the diagnosis of intravascular imaging. We describe the state of the art of developments and applications of PAI and OCT in intravascular imaging.

#### 2.1.1 Intravascular optical coherence tomography

Intravascular optical coherence tomography (IVOCT) is a fiber optic technology that is used for the evaluation of coronary lesion morphology and provides high-resolution (10-15 μm) cross-sectional images. Based on the principle of OCT, IVOCT detects an interference signal of light backscattered from the target tissue with the light backscattered from the reference mirror [46]. There are three main OCT methods: time-domain OCT (TD-OCT), Fourier-domain OCT (FD-OCT), and swept-source OCT (SS-OCT) [47]. The principle of TD-OCT is based on partial coherence interferometry and the change of optical path difference (OPD), which produces an A-scan profile [48]. In FD-OCT, the interferometer output is transmitted to an optical spectrometer. The fast Fourier transform of the spectrometer signal obtains the A-scan profile of the reflectivity in depth [47]. TD-OCT and FD-OCT both employ a wideband source, while SS-OCT uses laser sources. The SS-OCT uses the fast tunability of laser sources to label different time delays, which are then detected by interferometer [49]. The time of an A-scan depends on the time to tune the wavelength [48]. Similarly, there are two processing technologies that can be used to obtain intravascular OCT imaging: time-domain IVOCT (TD-IVOCT) and Fourier-domain IVOCT (FD-IVOCT). In IVOCT, TD-IVOCT mainly measures the alteration of interference signal derived by one light wavelength at a time. FD-IVOCT using Fourier transformation detects the interference signal of the whole spectrum at a single time point. Moreover, FD-IVOCT has the advantage of improved signal-to-noise ratio, which results in greater penetration depth without losing of vital detail or resolution [50]. OCT is used for intravascular imaging in the human body, which is mainly mediated by scattering. But blood and other tissues in the blood vessel increase significant attenuation. With the increase of wavelength, the attenuation of scattered light is suppressed, which can obtain the deeper and more distinct images of tissue. Near-infrared lasers are widely studied as light sources of IVOCT [51]. IVOCT images are obtained with a central wavelength range of 1250 to 1350 nm that enables an axial resolution of 10-15 μm and a lateral resolution of 20-40 μm at 3 mm. The IVOCT based on optical fiber and catheter is composed of a light source (“swept laser”), a detector, and a gradient-index lens with a microprism at the distal tip. The infrared light transmits from the light source via the optical fiber, then the distal lens and a microprism direct the beam, resulting in a focused output beam perpendicular to the catheter axis [50, 52].

In OCT, the central wavelength of light source determines the axial resolution, lateral resolution, and imaging depth. Since the optical properties of tissue depend on wavelength, an appropriate wavelength is chosen to maximize light penetration and enhance image resolution at deeper depths [53]. The first clinical application of the OCT system for ophthalmology uses near-infrared light of roughly 800-nm wavelength. In other applications, such as observation of intravascular tissue, 1.3-μm IVOCT systems have superior penetration depth compared with the 800-nm OCT system. However, the images of 1.3-μm IVOCT cannot directly reflect material composition, only obtaining information distribution of the scattering intensity. As a result, the accuracy of classification of plaque types is limited and the reliability of plaque diagnosis is affected by the proficiency of doctors. Recently, the long-wavelength window around 1.7 μm has been used for OCT imaging to enhance penetration depth due to the lower scattering in the tissues [54]. Tanaka et al. proposed a spectroscopic OCT using a light source at 1.7-μm band for imaging distribution of lipid in vascular plaque [55]. The spectroscopic OCT is based on the analysis of interference light spectrum, which can obtain information on the distribution of composition. The 1.7-μm OCT system demonstrated imaging of lipid distribution in an in-vitro artery model. In order to use better in human intravascular imaging, Li et al. presented a novel IVOCT system with a 1.7-μm center wavelength swept light source to obtain more structural information [56]. In addition, they compared the penetration depth of the 1.3- and 1.7-μm OCT systems in water and air, respectively. Therefore, the 1.7-μm OCT system holds great potential used for in-vivo applications of plaque characterization. Moreover, 2.1- and 4-μm OCT systems were proposed for deep tissue imaging to increase image depth and resolution [57-59].

A key technology for the application of OCT in intravascular imaging is a catheter that can transmit, focus, scan, and collect a single spatial-mode optical beam [60]. The intravascular catheters must be flexible and small in diameter to secure access to the entrance or ostium of the coronary arteries. The conventional OCT catheter consists of an optical coupling element at the proximal end, a single-mode fiber in the body, as well as an optical focusing and beam orientation elements at the distal end [61]. Currently, there are two types of catheters available commercially: the Ilumien optics Dragonfly imaging catheter and the Dragonfly optics catheter [62]. The Ilumien optics Dragonfly imaging catheters are intended for intravascular imaging of coronary arteries with a vessel diameter of 2.0-3.5 mm. The catheter can be automatically pulled back through the outer sheath of the tube and will not be exposed to any moving parts during the automatic imaging pullback. The Dragonfly optics catheter can be used for rapid exchange in imaging, and its tip tapers to a 2.7-F diameter, which can be compatible with other larger diameter catheters to provide rapid acquisition [62]. The catheter removes blood in the vessel during imaging process. Most of the probes have electric driven scanning device, especially the proximal-end scanning probes, which is susceptible to produce the image artifact. Lu et al. proposed a low-cost and shadow-free IVOCT catheter based on a miniaturized propeller that does not need an electrically driven scanning device [63]. Wang et al. firstly combined OCT catheter with fiber Bragg grating (FBG) to reconstruct the vascular shape and bending direction in real time [64]. The FBG-OCT catheter provides a visual image of the vessel, providing information for plaque measurements, blood flow velocity, and blood pressure. Kang et al. assembled a proximal-driven OCT catheter with a diameter of 0.86 mm [65]. The catheter features a type of all-fiber probe and a narrow nearly collimated pencil beam. The in vivo imaging capability of the catheter was demonstrated in animal samples. Moreover, for a more comprehensive assessment of intravascular disease, the design of complementary catheters for multimodal imaging has great clinical potential [66].

**Figure 2.**
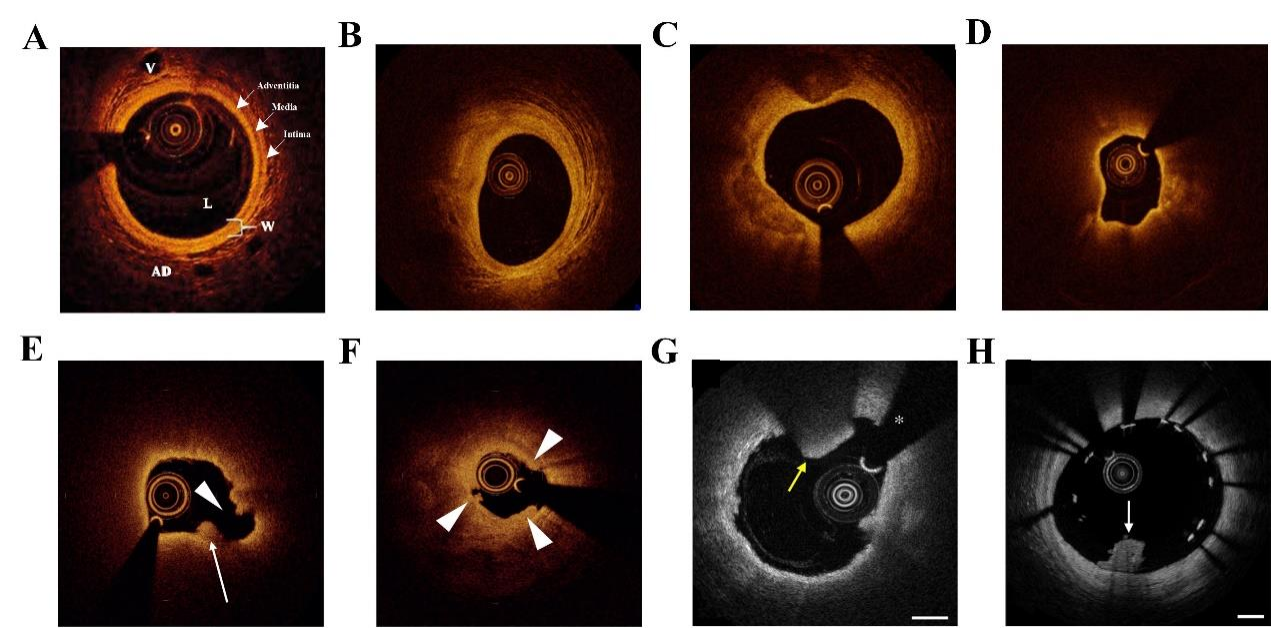
Clinical applications of intravascular optical coherence tomography. (A) normal coronary artery well. L: lumen; W: vessel wall; AD: adventitia; V: vasa vasorum; (B) fibrous plaque; (C) fibrocalcific plaque; (D) lipid-rich plaque; (E) plaque rupture; (F) plaque erosion; (G) red thrombus; (H) white thrombus. (A) Reproduced with permission from Ref. [46] Copyright 2015 Termedia; (B)-(D) Reproduced with permission from Ref. [68] Copyright 2012 Springer Nature; (E)-(F) Reproduced with permission from Ref. [23] Copyright 2018 The Korean Society of Cardiology; (G)-(H) Reproduced with permission from Ref. [70] Copyright 2012 Elsevier.

Many clinical trials have compared the histological characteristics of IVOCT and IVUS in coronary artery plaques, which display the advantage of IVOCT in visualization of the plaque composition due to its high resolution [23]. Currently, IVOCT is utilized in the diagnosis and evaluation of vascular diseases, including coronary plaque classification, acute coronary syndromes (ACS), and stent implantation. In IVOCT images, the normal coronary artery appears as a three-lay structure: three concentric layers have high/low/high scattering characteristics, corresponding to the intima, media, and adventitia, respectively (Fig. 2A) [46, 50, 67]. The IVOCT imaging offers a possibility to distinguish the various plaque components, such as fibrous plaque, fibrocalcific plaque, and lipid-rich plaque [68]. In IVOCT images, fibrous plaque is a homogenous tissue with high reflectivity and low attenuation (Fig. 2B). Fibrocalcific plaque manifests as sharply delineated, homogeneous, signal-poor region (Fig. 2C). Lipid-rich plaque has the diffuse border with a very rapid signal decay (Fig. 2D). Moreover, the punctate signal with high reflectivity and uneven shadowing might represent macrophages. IVOCT is able to show the histologic features of the vulnerable plaque precisely: a thin-capped fibroatheroma (fibrous cap thickness ≤65 μm) within a lipid-rich plaque [52]. In ACS, IVOCT is feasible in the vessel wall and plaque morphology, such as plaque rupture, plaque erosion, and intracoronary thrombus. Ruptured plaques show features of intimal tearing, disruption, or dissection of the fibrous cap and may form a cavity formation within the plaque (Fig. 2E) [23]. Plaque erosion is identified by an irregular luminal surface with the loss of endothelial lining but without cap rupture (Fig. 2F) [69]. IVOCT is also able to detect white and red thrombus in the arterial lumen about intracoronary thrombus. The red thrombus presents as an irregular structural cavity with low reflectivity and high attenuation of the signal, accompanied by a signal-free posterior shadowing due to the attenuation of red blood cells (Fig. 2G) [70]. While the white thrombus without red blood cells appears as an irregular structure with high reflectivity and low attenuation of the signal (Fig. 2H). In addition, IVOCT provides a detailed assessment of the implanted stents, such as lesion length, lesion severity, landing zone, and stent sizing. It assists the pre-procedural planning with exact dimensions, detection of complications and facilitates the operators to achieve optimal treatment results [67].

**Figure 3.**
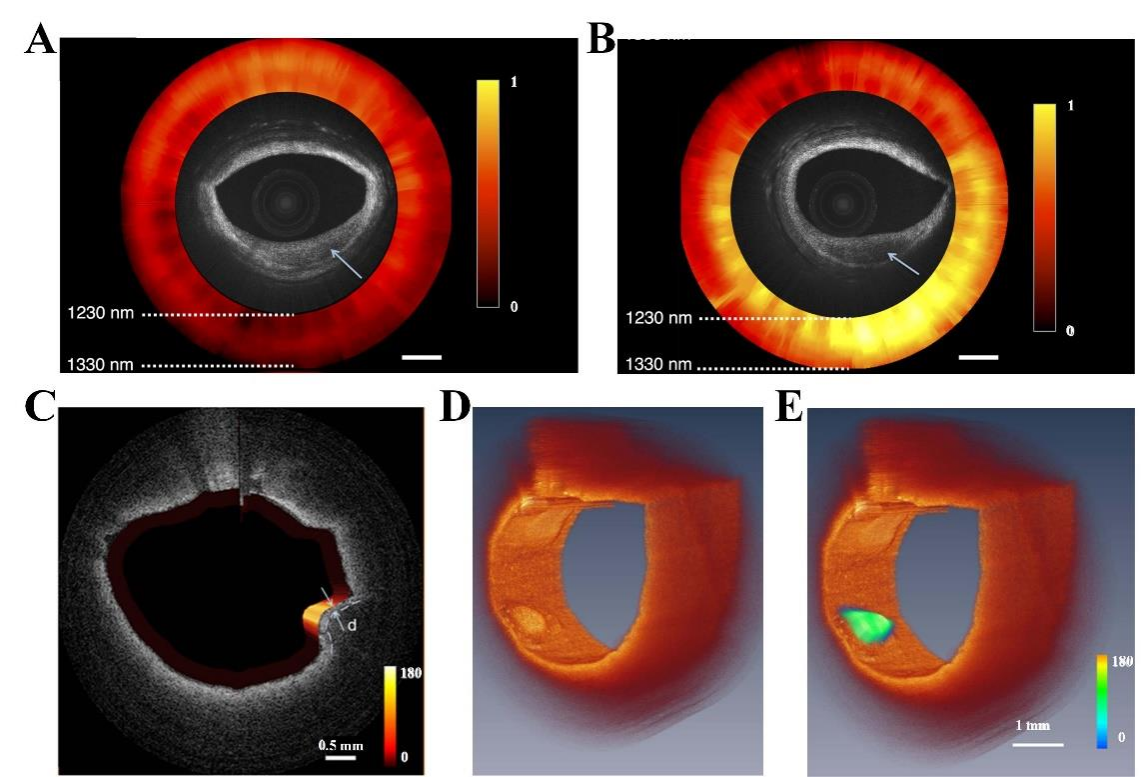
Images of intravascular plaques based on OCT-NIRS and OCT-NIRF. (A) low lipid signal of the OCT-NIRS image. (B) high lipid signal of the OCT-NIRS image. (C) the fused fluorescence-OCT image, (D) 3D OCT image, and (E) 3D OCT image combined with two dimensional NIRF image. (A)-(B) Reproduced with permission from Ref. [75] Copyright 2013 Optical Society of America (OSA). (C)-(E) Reproduced with permission from Ref. [79]. Copyright 2012 Society of Photo-Optical Instrumentation Engineers (SPIE).

Moreover, the addition of optical techniques will increase the effective information of plaque images and enhance the accuracy of plaque diagnosis, such as near-infrared spectroscopy (NIRS) and near-infrared fluorescence (NIRF) [71]. NIRS can obtain detailed analysis of chemical composition in the wavelength range of 400-2400 nm, which is based on the absorbance and scattering of light by biological specimens [72]. NIRS can identify lipid-rich atherosclerotic plaques and assess vulnerability of plaques [73]. However, the accurate depth of lipid in the vessel wall cannot be identified by NIRS [74]. Intracoronary NIRS and IVUS are always combined to obtain co-registered structural and chemical information [73]. The light of OCT system is also used to provide NIRS data due to the overlap of bandwidth between OCT light source and absorption features of lipid [71]. Catheter-based OCT-NIRS data collected from cadaver coronary arteries showed that these two technologies could acquire information of microstructural and compositional simultaneously [75]. Moreover, OCT-NIRS can discriminate lipid signal in two plaques that are similar in appearance through the color of spectrum, as shown in Fig. 3A. The NIRF is an emerging molecular imaging modality at the wavelength of 700-1000 nm, which provides an extremely high signal/background ratio with the appearance of tissues not be changed [76]. Compared with visible light, NIRF can achieve deeper penetration into tissues by using fluorescence and obtain lower background of autofluorescence in nontargeted tissues [77]. Moreover, intravascular NIRF catheters appear high transferability that can be used to the cardiac catheterization laboratory, thus offer a new method in vivo to detect atherosclerosis and assess high-risk coronary plaques [78]. Therefore, the combination of three-dimensional (3D) OCT with NIRF, a clinical high-resolution structural imaging approach, can obtain both molecular and morphological information, as well as quantitative NIRF image [79]. As shown in Fig. 3B, OCT-NIRF system can detect both OCT and NIRF images simultaneously, and the OCT image of plaque matches well with the two-dimensional fluorescence intensity image. Indocyanine green (ICG) is an amphiphilic NIRF imaging agent used in OCT-NIRF catheter, which shows detailed imaging of lipid-rich inflamed plaque [80]. The intracoronary OCT-NIRF imaging with a clinical dose of ICG was feasible to accurately assess inflammation related to plaque and DES in coronary artery [81, 82]. Li et al. presented a tri-hybrid intravascular imaging system with IVUS, OCT, and NIRF technologies, indicating the potential in multi-structural and molecular imaging [83]. The diameter of tri-hybrid intravascular catheter is 1 mm, which means that the catheter can be applied in imaging and diagnosis of intravascular lipid plaques.

**Figure 4.**
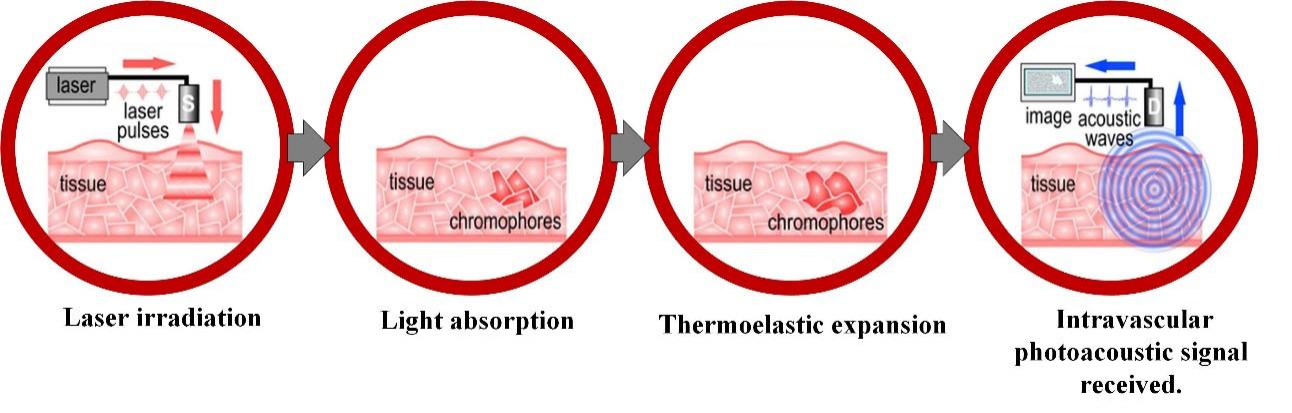
Schematic diagram of intravascular photoacoustic (IVPA) imaging. Reproduced with permission from Ref. [85] Copyright 2019 Elsevier.

#### 2.1.2 Intravascular photoacoustic imaging

Intravascular photoacoustic (IVPA) imaging is a newly developed catheter-based imaging technique for the diagnosis of intravascular diseases, which combines the advantages of ultrasonic and optical imaging. The IVPA catheter is integrated with a multimode optical fiber, an optical reflector, and an ultrasonic detector, which is pushed to the distal end of the target vascular lumen during imaging. A probe at the catheter tip emits short laser pulses for a few nanoseconds to irradiate the arterial wall. The pulses energy is absorbed by the surrounding tissues, then converted into heat and caused thermal expansion (Fig. 4) [84, 85]. The transient pressure rise excites a wide band of ultrasonic waves that are collected by the ultrasonic detector. The distribution of absorbed light energy density is reconstructed by an inversion algorithm [86]. Depending on the specific optical absorption characteristics of different tissues, IVPA imaging can use specific laser wavelengths to obtain chemical characterization of vessel wall components to identify vulnerable plaque. Due to the low blood absorption coefficient and deep penetration depth of the near-infrared wavelength, the 1210 and 1720 nm bands are considered as the most suitable bands for IVPA imaging of plaques. To achieve in vivo imaging, a miniature and highly sensitive catheter is an essential element to enter blood vessel. A typical IVPA catheter consists of an optical fiber for light delivery and an ultrasonic transducer for receiving signals, therefore, IVUS images are collected with IVPA images simultaneously. This provides anatomical images that are automatically co-registered with the photoacoustic image and offers both advantages of broad imaging depth of IVUS and composite contrast of IVPA [87]. IVPA/US imaging is a powerful and promising alternative to identify vulnerable plaque [88].

The development of IVPA imaging is driven by the availability of suitable light sources. In the early stage, the IVPA imaging of human atherosclerosis and lipids focused on the visible wavelength range (410-680 nm), however, blood absorption coefficient is very high at these wavelengths [89]. The introduction of near-infrared light sources promotes the study of IVPA imaging and increases the penetration depth. Besides, blood absorption coefficient is low in the wavelength range from 680-1300 nm. The lipid absorption spectrum exhibits a large peak around 1210 nm, which is used to distinguish lipids from other tissue components of blood vessel walls [90]. In addition, Anderson et al. measured the absorption spectra of human fat, identifying promising bands near 1210 and 1720 nm [91]. Wang et al. found that the stimulated photoacoustic signal at 1730 nm was five times stronger than that at 1210 nm [92]. To achieve accurate and high-speed IVPA imaging, a nanosecond-pulsed and wavelength-tunable laser is critical. The laser source needs to be considered two interplay parameters: laser pulse energy and repetition rate. In practical IVPA imaging systems, laser pulse energy reaches to several tens of micro-joules to generate signals. The previous laser source mainly employed optical parametric oscillators (OPOs) with the highest pulse repetition rate of 5 kHz [93-95]. Moreover, the expensive and cumbersome OPOs limit the imaging speed and increase the cost of IVPA imaging. Fiber lasers are excellent candidate for the alternative source due to its compact size, low cost, and high tunability. Sang et al. presented a wavelength-switchable nanosecond-pulsed erbium-doped fiber laser (EDFL) based on the active mode-locking technique [96]. The pulse repetition rate of the EDFL can be tuned from 200 kHz to 1 MHz, and the pulse energy is within the hundred-nanojoule range to fulfill the requirements of IVPA imaging. Li et al. reported a high-energy gain-switched thulium-doped fiber laser (TDFL) for PAI of lipids at 1700, 1725, and 1750 nm [97, 98]. In the three laser wavelengths, the pulse energies of TDFL are 58.2, 66.8, and 75.3 μJ, respectively, with a 16.7 ns pulse duration at the repetition rate of 10 kHz.

**Figure 5.**
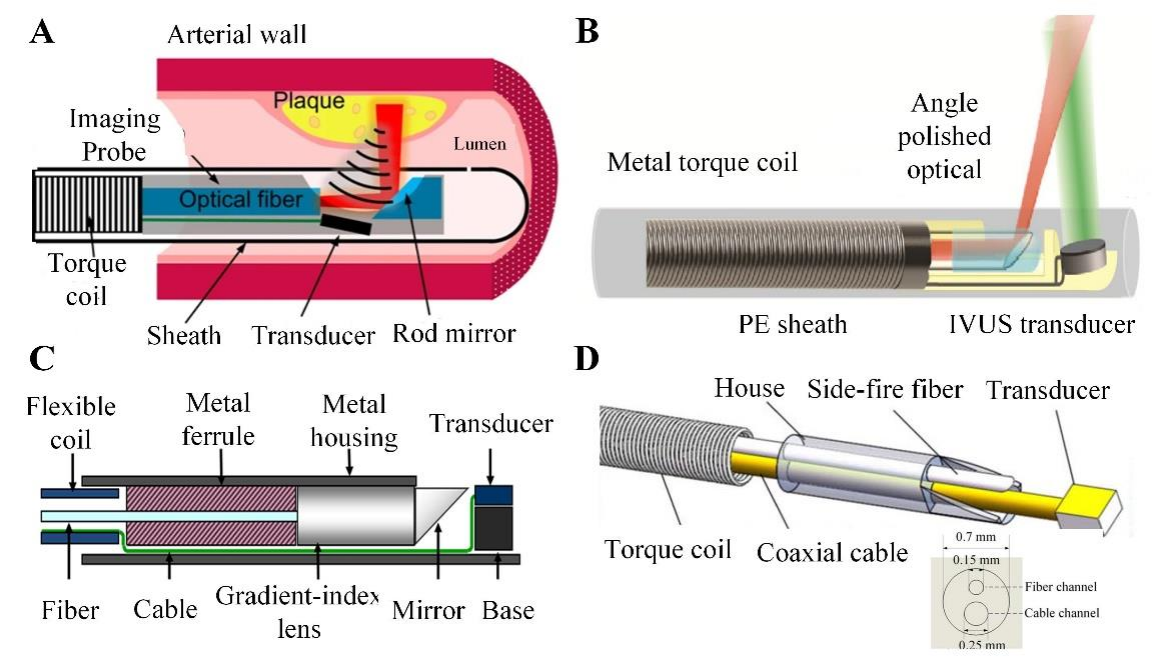
Designs of intravascular photoacoustic (IVPA) imaging catheter. (A) collinear IVPA catheter; (B) the longitudinally offset dual-beam catheter; (C) the IVPA catheter of 0.9 mm outside diameter; (D) the ultrafine imaging catheter with a diameter of 0.7 mm. (A) Reproduced with permission from Ref. [99] Copyright 2018 Springer Nature; (B) Reproduced with permission from Ref. [95] Copyright 2017 Optical Society of America (OSA); (C) Reproduced with permission from Ref. [94] Copyright 2015 Society of Photo-Optical Instrumentation Engineers (SPIE); (D) Reproduced from Ref. [101] Copyright 2019 Optical Society of America (OSA).

The IVPA imaging catheter is suitable for clinical conversion of coronary arteries to satisfy the requirements: miniature, flexibility, safety, and compactness [45]. A typical IVPA imaging catheter mainly consists of a light delivery part, an acoustic transducer, a torque coil, and an outer sheath. There are mainly two types of IVPA catheter design: a collinear IVPA catheter and an IVPA catheter with a longitudinal offset between optical output and the acoustic transducer. The collinear catheter allows for complete overlap of optical and acoustic beams, accomplishing a high signal sensitivity (Fig. 5A) [99]. In the longitudinally offset dual-beam catheter, the optical port is positioned proximal to the ultrasound transducer. The light beam is emitted from a conduit at an angle to illuminate the tissue in front of the transducer (Fig. 5B) [95]. Song et al. reported that the IVPA catheter external diameter was 0.9 mm in 2015, which was below the 1 mm threshold for clinical conversion to coronary arteries (Fig. 5C) [94]. Recently, the IVPA system achieved the imaging speed as high as 100 frames per second based on the 0.9-mm catheter [100]. Lei et al. achieved the IVPA system with the lateral resolution of 13 μm and the ultrafine imaging catheter with a diameter of 0.7 mm (Fig. 5D) [101, 102]. Fiber optic technologies take advantages of the flexibility and compactness of the optical fiber, which facilitate the miniaturization of catheter size [45]. Wang et al. reported a 1-mm catheter based on a tapered fiber, which made the IVPA obtain the lateral resolution of 18 μm and the axial resolution of 31 μm [103]. Cao et al. presented a dual-frequency catheter with the diameter of 1 mm. In this catheter, the low-frequency transducer enhances photoacoustic sensitivity and the high frequency transducer maintains spatial resolution for ultrasound imaging [104]. In addition, Leng et al. demonstrated a novel tri-modality imaging system that can achieve 3D intravascular imaging through a 0.9-mm miniature catheter [66].

**Figure 6.**
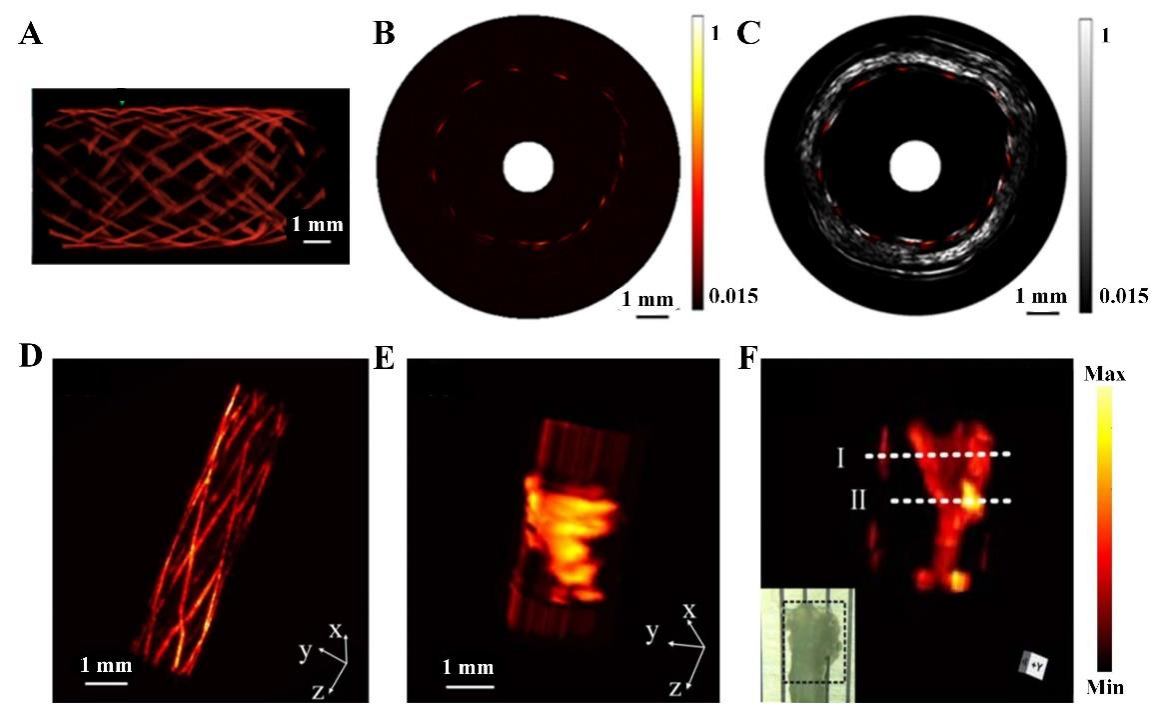
IVPA images of stent and lipid with different diameter catheters. (A) 3D IVPA image, (B) IVPA image, and (C) IVPA/US cross section images of a stent detected by 0.9-mm catheter. (D) Reconstructed 3D IVPA image of stent, (E) PA image of the fine vessel with lipid core, and (F) Reconstructed 3D IVPA image of lipid detected by 0.7-mm ultrafine catheter. (A)-(C) Reproduced with permission from Ref. [94] Copyright 2015 Society of Photo-Optical Instrumentation Engineers (SPIE). (D)-(F) Reproduced with permission from Ref. [101] Copyright 2019 Optical Society of America (OSA).

In order to introduce IVPA imaging into clinical practice, PAI can be integrated with the IVUS imaging system. Both IVPA and IVUS imaging systems can utilize the same ultrasonic sensor and relevant receiving electronic devices. Moreover, IVPA/US can provide tissue composition and morphology simultaneously. Many studies have shown that it is possible to detect lipids in atherosclerotic plaques using combined IVPA/US imaging in vivo [105, 106]. Experimental simulations have been conducted in animals and humans [105-107]. Recently, Wang et al. displayed the distribution and the relative concentration of lipid by the tapered fiber-based IVPA/US imaging catheter, which provides more parameters for the accurate assessment of atherosclerotic plaque [103]. Lei et al. assessed the reliability of the IVPA/US imaging of lipid by a parametric study [102]. The sensitivity, specificity, and accuracy of the IVPA/US imaging were 96.8%, 83.3%, 94.6% and 97.3%, 72.7%, 95.2%, respectively, in rabbit abdominal aorta and human plaque. These data provide a great opportunity for IVPA/US imaging to accurately diagnose intravascular plaques in the clinic. The IVPA/US imaging system is also suitable for imaging of metallic implants in vivo [108]. Li et al. used photoacoustic and ultrasound to image intravascular stents in healthy rabbits, demonstrating the potential of IVPA system for intravascular imaging [94]. As shown in Fig. 6A, the fine structure of the stent is clearly visualized by 0.9-mm diameter catheter. Moreover, the IVPA/US image can realize high-contrast imaging for the structure of vessel and stent. It can be summarized that the IVPA/US imaging system is superior to the single imaging modality for guiding and evaluating stenting. Lei et al. used a 0.7 mm-based ultrafine catheter for IVPA/US imaging to obtain the 3D images of stent and the fine vessel-mimicking sample with a lipid core [101]. Fig. 6B exhibits the high-contrast 3D images of stent and lipid, which demonstrates the reliability and feasibility of the ultrafine IVPA catheter.

#### 2.1.3 The combine of IVOCT and IVPA imaging

The OCT and PAI technologies are complementary in imaging depth, imaging resolution, and imaging speed, which promote them a suitable companion for a multimodal imaging system (see Table 1). In intravascular PAI-OCT, the IVPA imaging system is able to penetrate deep tissues and provide molecular information about plaque composition, while IVOCT maintains high resolution and depth resolution scattering contrast for lipid-rich plaques [26]. IVPA imaging integrated with IVUS provides both structure and chemical compositions of arterial walls, which demonstrates the capability of characterizing vulnerable plaque [99]. The combination of IVUS and OCT imaging systems (IVUS-OCT) could provide high resolution and large penetration depth, visualizing the plaque structural information on different spatial scales [109]. The tri-modality IVOCT-PA-US provides depth-resolved molecular contrast and morphology of coronary arteries without the use of a contrast agent, which has been demonstrated and tested in human arteries ex vivo [110]. Recently, Leng et al. reported the tri-modality imaging system that can perform 360° continuous rotation and pull-backing with a 0.9-mm miniature catheter [66]. The tri-modality imaging system was performed in ex vivo animal sample experiments, which obtained the merged tri-modality images. In addition, future direction for multimodal intravascular imaging studies could be focused on improving scanning speed, miniaturizing the probe size, and further optimizing detection performance.

**Table 1:** Comparison of intravascular optical coherence tomography and intravascular photoacoustic imaging.

Imaging parameters	Intravascular Optical Coherence Tomography	Intravascular Photoacoustic Imaging
Contrast mechanism	Scattering	Absorption
Axial resolution	Determined by the spectral bandwidth, or range of wavelengths in light source (typically approximately 15-20 μm).	Depending on the detected photoacoustic bandwidth (typically approximately 30-40 μm).
Lateral resolution	Determined by the central wavelength of the light source and imaging optics (typically approximately 20-40 μm).	Depending on the implementation range from 400-500 μm.
Laser wavelength	1.3, 1.7 μm	1.2, 1.72 μm
Imaging depth	Restricted by the optical transport mean free path, and usually ranges from 0.1-2.0 mm.	Depending on the implementation approximately 3.0 mm.
Imaging speed	Defined by the sweep rate of laser.	Defined by the laser pulse repetition rate, mechanical scanning speed, or the multiplexed data acquisition time.
Ref.	[39, 50, 52, 56, 70]	[45, 84, 85, 105]

### 2.2 Laser technologies for intravascular treatment

The characteristics of laser are the bases for therapeutic applications, such as monochromaticity, coherence, collimation, and high-power density. Lasers with different wavelengths are applied precisely to specific tissues. The power density is represented by four parameters: energy, power, fluence, and irradiance [111]. The energy is measured in joules, and the power is consumed in watts. The fluence refers to the energy density of a laser beam in joules per square centimeter, while the irradiance refers to the power density of a laser beam expressed as watts per square centimeter. By manipulating the laser parameters, people use lasers to act on biological tissues based on specific clinical purposes. The mechanism of laser-tissue interaction may include thermal, photoablative, photochemical, and/or photodisruptive effects. These effects have been used for intravascular treatment [112]. Thermal effects can be generated by absorption of laser energy, direct laser ablation, and laser-heated catheter tips. Thermal effects in angioplasty produce thermal injury, which causes vasospasm and ultimately restenosis. Photochemical effects refer to the bond-breaking mechanism, and the photodisruptive effect is the use of shock-wave disruptive laser angioplasty to produce particulate debris in sizes ranging from microns to hundreds of microns. Laser technology is widely used in the treatment of vascular diseases based on its characteristics and laser-tissue effects. Laser angioplasty is suitable for the treatment of intra-arterial thrombosis to achieve recanalization, including excimer laser (308 nm) and third harmonic of Nd:YAG laser (355 nm). EVLA is used for the treatment of varicose veins, resulting in fibrosis and occlusion of the treated vein.

#### 2.2.1 Laser angioplasty

The excimer laser is a kind of pulsed gas laser, which uses a mixture of rare gas and halogen as the active medium to generate short-wavelength and high-energy ultraviolet pulses at the wavelength of 308 nm [113]. Compared with lasers emitted in infrared range, the excimer laser has the advantage of a smaller absorption depth (<100 μm), reducing the risk of tissue damage. Excimer lasers can be used in clinical disease treatment owing to the property of “cold laser”. The penetration depth of excimer lasers is shallow, which ensures accurate ablation of tissue without generating too much heat and minimizes inadvertent tissue damage. Excimer laser angioplasty is mediated by three different mechanisms: photochemical, photothermal, and photomechanical [114]. Photochemical action does not generate heat and excimer laser energy is larger than the binding energy of molecular bonds, which leads to the fracture of molecular mechanical bonds. Laser radiation is absorbed by tissues due to the photothermal effect and converted into thermal energy. During the absorption process, the molecular bonds are also vibrated to generate heat, and water evaporates from cells, causing them to burst and create bubbles of steam. The photomechanical effect refers to short and high-energy pulses of radiation causing local formation of the plasma and generating mechanical shock forces. Rapid expansion and collapse of steam bubbles further break plaques, removing the by-products (water, gas, and small particles) of ablation.

In excimer laser, fluence refers to the threshold energy required for ultraviolet light to penetrate tissues and produce vapor bubbles, with the range of 30-80 mJ/mm^2^. The number of pulses emitted per second is the pulse repetition rate, and the duration of each pulse is termed a pulse width. The current excimer laser (CVX-300) is approved for the treatment of CAD, known as excimer laser coronary angioplasty (ELCA). The system uses the catheter output fluence generated between 30-80 mJ/mm^2^ with pulse repetition of 25-80 Hz and pulse width of 125-200 ns. The major advantage of laser catheters is that it is compatible with any standard 0.014-inch guidewire. The ELCA catheters are designed in four diameters: 0.9, 1.4, 1.7, and 2.0 mm, the 0.9 mm ELCA catheter is sufficient for the treatment of CAD [115]. The 0.9-mm catheter has the ability to emit high power (80 mJ/mm^2^) and laser energy at the highest repetition rate (80 Hz). Based on the arrangement of laser fibers on the tip of catheter, the laser catheter includes two types: concentric catheter and eccentric catheter. Concentric catheters are commonly used for intravascular laser treatment, while eccentric catheters are recommended for ablation of eccentricity lesions such as in-stent restenosis (ISR) and bifurcation lesions. The saline-infusion technique allows laser to enter the tissue from the tip of the catheter without any interference, which reduces the risk of dissection. During the treatment, laser catheter was pushed at a rate of 0.5 mm/s and the saline was injected at a rate of 2-3 ml/s [116].

ELCA has been studied in different types of coronary artery lesions, such as ACS, noncrossable/nondilatable lesions, chronic total occlusions (CTO), ISR, saphenous vein grafts, and so on. Here, we mainly discuss the treatment of three types of disease: ACS, ISR, and CTO.

**Figure 7.**
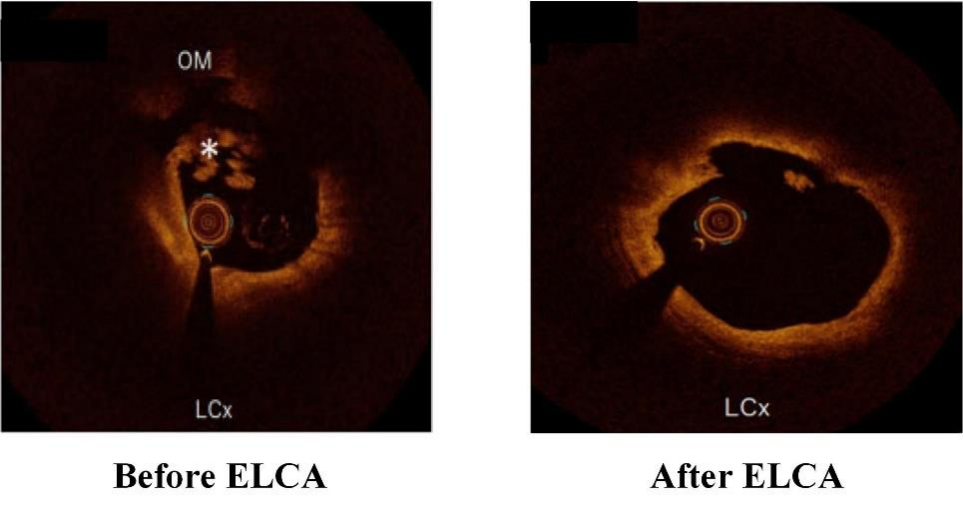
The changes of ELCA before and after operation were shown by OCT. Reproduced with permission from Ref. [120] Copyright 2020 Oxford University Press.

The interaction between ultraviolet beam in 308 nm and platelets has demonstrated a phenomenon of altered aggregation kinetics, which is manifested by decreased platelet aggregation and reduced platelet force development [117]. The excimer laser has the capacity of evaporating thrombus, suppressing platelet aggregation, and ablating potential plaques, which has proved to be effective. In patients with ACS, the presence of thrombus increases the incidence of major adverse cardiac events and infarct-related artery stent thrombosis. The advantages of ELCA for ACS are the rapid thrombus removal with the vaporization of procoagulant reactants and the reduction of risk of distal embolization [118]. In some patients with ACS, including AMI or unstable angina pectoris, it is feasible and safe to use an excimer laser. Recently, Shibata et al. added data on the effect of ELCA on myocardial salvage in patients with acute ST-segment elevation myocardial infarction (STEMI), which used radionuclide imaging to evaluate the results [119]. Their nuclear scintigraphy results showed that ELCA was superior in saving patients with the first attack of STEMI, there were no complications associated with ELCA. Moreover, ELCA can safely reduce intracoronary thrombus with the guidance of OCT [120]. The OCT images showed changes in the minimum lumen area before and after the ELCA procedure (Fig. 7).

Despite advances in angioplasty and the existence of high-pressure balloon catheters, handing newly deployed coronary stents remains a technical challenge. ISR is still a complication after stent implantation with high relapse rate. It was reported that 10%-50% of patients who received bare-metal stents suffered ISR [121]. Recently, the ELCA is thought to be advantageous for ISR treatment. Hirose et al. showed that ELCA is relatively safe and feasible for the treatment of ISR compared with balloon dilatation alone [122]. Compared with bare-metal stents, the introduction of DES greatly reduces angiographic restenosis and target vessel revascularization [123]. Hajibandeh et al. proposed a combination of DES, ELCA, and standard balloon angioplasty for the treatment of ISR, and the clinical effects have been greatly improved [124]. The paclitaxel drug-coated balloon (DCB) and ELCA have been shown to be safe and efficient alternatives in the current treatment of ISR [125, 126]. The experiments showed that the effect of utilizing DCB alone is inferior to that of ELCA with DCB treatment. The ISR treatment using ELCA guided by OCT is a safe and feasible method with high success rates [127, 128]. Although ELCA is an effective device for the treatment of ISR, it may require a large amount of ELCA energy to obtain optimal outcomes.

Among some of these difficult lesions, calcified stenosis, CTO, and non-compliant plaques remain major technical challenges [129]. In recent years, ELCA has gradually been applied to CTO, which could open channels for CTO lesions at the molecular level and change the compliance of lesions before direct balloon angioplasty [130]. However, in calcified lesions, the effective rate of ELCA is the lowest. Rotational atherectomy (RA) has become a treatment derived from ELCA, which remains the cornerstone for the treatment of heavily calcified lesions and is currently the first choice for severely stenosed calcified lesions [131]. Although RA can be performed in the presence of severe calcification, it is contraindicated in AMI, while ELCA is highly useful in acute settings. Recently, ELCA combined with RA in severely calcified lesions has been described as “RASER” [132, 133]. The clinical effect of excimer laser combined with traditional PCI, RA, and DCB is better than that of a single method. Moreover, experts conducted experiments and evaluated clinical effects. The results show that ELCA combined with other methods have a good clinical effect in the treatment of severe and complex coronary arteries.

Although ELCA has the advantages of reducing dissection, perforation, and adverse complications, it also has some inherent drawbacks. Primarily, in ELCA, patients are injected with significant amounts of contrast agent, and the accumulation of iodine-based solution may result in renal complications. Furthermore, ELCA has a poor ablation effect on calcified plaques, limiting the use of laser therapy in calcified vascular disease. Recently, Herzog et al. proposed a solid-state laser at a wavelength of 355-nm (third harmonic of Nd:YAG) used for the treatment of thrombotic stroke and PAD [134, 135]. The nanosecond pulses at the wavelength of 355 nm under the guidance of optical fiber caused only slight damage to specimens immersed in contrast medium, but no damage immersed in normal saline [134]. Then, the 355 nm laser catheter was used for the treatment of PAD, and particularly for calcified occlusions [135]. The laser catheter is a hybrid catheter comprising optical fibers for the delivery of 355 nm laser pulses at 30 Hz and 6 J/cm^2^, combined with a blunt mechanical blade [136]. The B-Laser™ atherectomy system (Eximo Medical, Israel) was approved by the Food and Drug Administration. We compare the two laser systems (Excimer laser system and B-Laser™ atherectomy system) in Table 2. John et al. and Shammas et al. evaluated the treatment of PAD with the B-Laser™ atherectomy system, which is effective and safe in ablating atheroma and calcified plaque [137, 138]. Alperovich et al. first proposed the use of a 355-nm laser device with a step-by-step approach to classify ablated tissue utilizing sound signals recorded by a microphone placed nearby [139]. This technique can be used to identify the correct location in the vasculature, thereby further reducing the risk of perforation during 355-nm laser atherectomy.

**Table 2:** The comparison of excimer laser system and B-Laser™ atherectomy system.

Parameters	Excimer Laser system	B-Laser™ system
Active medium	XeCl	Nd:YAG
Wavelength	308 nm	355 nm
Catheter sizes	0.9, 1.4, 1.7, 2.0 mm	0.9, 1.5, 2.0, 2.35 mm
Catheter output fluence	30-80 mJ/mm^2^	50-60 mJ/mm^2^
Pulse repetition rate	25-80 Hz	40 Hz
Pulse width	125-200 ns	10-25 ns
Weight	295 kg	85 kg
Length	114 cm/45 in	74 cm/29.13 in
Height	89 cm/35 in	95 cm/37.4 in
Width	61 cm/24 in	33 cm/13 in
Ref.	[113, 114, 118]	[135, 137, 138]

#### 2.2.2 Endovenous laser ablation

EVLA, a minimally invasive treatment for varicose veins of the lower limbs, has been used more frequently in recent years [140-143]. Varicose veins are caused by reflux of blood due to weakened connective tissue and insufficiency of venous valves. The consequence of varicose veins widens the great saphenous vein (GSV) and small saphenous vein (SSV). In the process of laser-tissue interaction, the predominant action mechanism of EVLA is photo-thermolysis [141]. Guided by ultrasound, a catheter is inserted into the vessel and a laser fiber is introduced into the lumen, where light energy is emitted. The energy is absorbed by blood, water, or vein walls in the lumen, leading to an increase in vascular temperature, which causes endothelial damage and collagen denaturation [144]. EVLA causes thermal damage to the venous wall and fibrotic occlusion of the GSV, obliterating the refluxing vein irreversibly [140]. In ELCA treatment, operational parameters could affect the therapeutic effect such as laser wavelength, laser output, laser energy, and fiber tip type.

In EVLA, different laser wavelengths (810, 940, 980, 1064, 1320, 1470, 1510, and 1920 nm) generate thermal energy, ultimately causing fibrosis and occlusion of the treated vein. Based on the absorption characteristics of vascular tissues, laser wavelengths in the near-infrared spectrum can be divided into hemoglobin-specific laser wavelengths (HSLW) and water-specific laser wavelengths (WSLW). Hemoglobin and myoglobin in vein wall smooth muscle components are the main chromophore at the laser wavelength of 810-1064 nm, while water within in the vessel wall is preferentially absorbed in 1320 nm and longer wavelengths [140]. In laser therapy, fluence (J/cm^2^) is used to quantify the amount of laser energy given per unit area. However, the surface area of the venous wall (cm^2^) is difficult to estimate. Current studies report linear endovenous energy density (LEED) as a surrogate marker of fluence. The LEED (J/cm) is measured by the total energy used (J) divided by the total length of vein treated (cm) [34]. The appropriate level of LEED in the range of 60-80 J/cm is safe and effective for the treatment of varicose veins. Longer wavelengths targeting water might have a lower optimal LEED (30-50 J/cm). Once the level of LEED exceeds 95 J/cm, especially above 100 J/cm, complications such as pain and paresthesia increase [145]. In addition, the LEED could be influenced by vein diameter and fiber tips.

**Figure 8.**
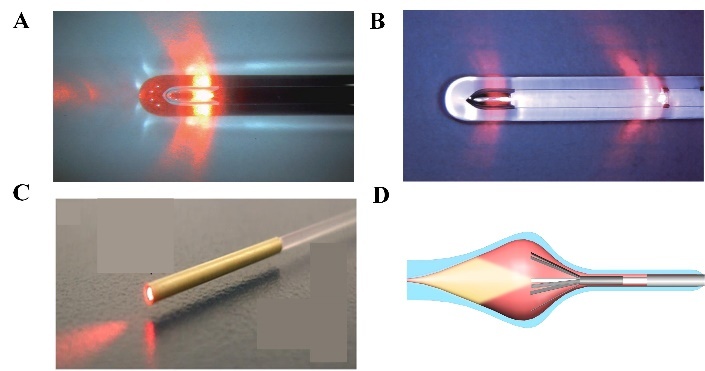
Types of the fiber tip. (A) The radial fiber; (B) The double-ringed radial fiber; (C) The neverTouch fiber; (D) The tulip fiber. (A) Reproduced with permission from Ref. [146] Copyright 2014 Annals of Vascular Diseases. (B) Reproduced with permission from Ref. [147] Copyright 2015 Annals of Vascular Diseases. (C) Reproduced with permission from Ref. [155] Copyright 2019 Springer Nature.

The original lasers used bare-tip fibers, and direct contact of the bare-tip fibers with the vein wall may result in the perforation of vein wall and increase pain and bruising. Some fiber tips other than bare lasers have resulted in significant reductions in pain and bruising as well as reduced ulceration and perforation, including radial fiber, double-ringed radial fiber, the neverTouch fiber, and tulip fiber. The radial fiber has a quartz tip at the distal end, reflecting the laser in a radial direction to disperse the emitted energy. The radial (360°) energy emission ensures homogenous photothermal destruction of the vein wall, allowing immediate closure of the vein (Fig. 8A) [146]. The double-ring radial fiber refers to the proximal irradiation of fiber optics contracting vein in advance, then the distal irradiation ablating the vein wall reliably (Fig. 8B). Moreover, since the laser is irradiated in two doses, the energy density is approximately half of that of the radial fiber, preventing the adhesion of the vein wall [147]. The neverTouch fiber with a coated tip or “jacket-tipped” eliminates laser tip contact with the vein wall (Fig. 8C). The neverTouch fiber minimizes pain and bruising caused by perforations of the vein wall compared to traditional bare-tip fibers. The jacket-tip also maximizes ultrasonic visibility, making it easier for physicians to use. Tulip fiber is a bare fiber with a tube at the end with self-expandable blades, and the tip of fiber is located inside the tube (Fig. 8D). The angle of light divergence is 44°, which avoids direct contact between the fiber tip and vein walls [142]. Currently, radial fiber and double-ring radial fiber are commonly used in EVLA treatment, while tulips are rarely used for varicose veins.

Under ultrasound imaging guidance, a guidewire passes through the hollow needle into the vein lumen. Then, the needle is withdrawn, and a small incision is made at the entry point of the guidewire to permit the passage of the introducer sheath and the dilator over the wire. Subsequently, the guide wire and the dilator are removed, leaving the sheath in place to allow the laser fiber to pass through. The correct location of the laser fiber tip is 2 cm away from the connective tissue, or just below the superficial inferior abdominal vein [148]. During the treatment of the GSV, the laser fiber tip has a safe distance from the femoral vein to avoid major complications such as deep vein thrombosis. In the treatment of SSV, the laser fiber enters from the middle to lower third of calf, with the same precautions for more peripheral access due to the potential for injury to the sural nerve [143]. To achieve the uniformity of EVLT-induced thermal damage to the vein wall, the vein is compressed only under the influence of tumescent anesthesia until the venous wall is exposed to the surface of fiber. Wendy et al. reviewed the available randomized controlled trials of EVLA efficacy and concluded that the success rate of ELCA was as high as 92% [149]. Pavei et al. analyzed data on the status of patients at least 9 years after EVLA. Experimental data suggested that EVLA with a 1470-nm laser diode and radial fibers provides stable and valuable long-term outcomes in patients with either GSV or SSV insufficiency [150]. However, EVLA is not a unique surgical method of treatment, and the complication rate in patients was 4.87% of EVLA procedures [151]. An unintended complication of EVLA is endothermal heat-induced thrombosis of the common femoral vein. Therefore, Hesham et al. recommended venous ultrasound examination at 2 days and 6 weeks after EVLA surgery to confirm saphenous vein ablation and absence of thrombus propagation to the common femoral vein [152].

In EVLA, most complications can be avoided by developing a standard regulation on their detection and treatment as early as possible, such as deep vein thrombosis, pain syndrome, and fragmentation of the tip of the radial fiber [151]. Besides, ultrasound imaging has limitations for tracking the accurate location of the fiber tip inside small veins such as angular dependency and comet tail artifacts. PAI, a non-invasive high-resolution imaging technique with high intrinsic contrast thermal correlation changes, is a promising tool for providing online feedback during EVLA [153]. Due to the similarity between PAI and EVLA in the catheter, the choice of PAI for tracking the fiber does not require major change in existing ablation catheters. The combination of ultrasound and PAI also has the advantage of real-time monitoring of temperature rise during laser ablation [154]. Yan et al. proposed to carry a low-power pulse laser beam on the laser-ablated fiber so that photoacoustic signals could be generated at the interface between the fiber tip and the tissue [155, 156]. The combination of ultrasound and PAI could be used to accurately guide the ablation fiber into the perforated vein and monitor the position of the fiber within the tip in the vessel. In addition, the PAI system was used to monitor the temperature inside the vascular lumen in real time by placing an optical fiber coupled with a pulsed laser beam in the catheter. The catheter tip tracking and temperature monitoring capabilities of the PAI-guided EVLA system were validated in vivo using a canine model [156].

#### 2.2.3 Laser angioplasty and EVLA

Depending on the therapeutic purpose, laser technology for the treatment of intravascular diseases includes intravenous therapy and intra-arterial therapy. Essentially, treatment of arterial vessels requires grinding clots into small particles in vessels to recanalize the vessels, while endovascular treatment requires fibrosis and occlusion of the vessels through thermal effect to ensure no reflow. Therefore, the laser parameters required by the mechanism of action and the vascular lesions targeted will also be different during treatment (see Table 3).

**Table 3:** Laser technologies for intravascular therapy.

Parameters	Laser angioplasty	Endovenous laser ablation
Laser wavelengths	308 nm; 355 nm	HSLW: 800-1064 nm; WSLW: 1320-2000 nm
Action mechanism	Photochemical; Photothermal; Photomechanical	Photo-thermolysis
Chromophore	DNA/RNA	Hemoglobin, Water
Fluence	3-8 J/cm^2^	15-100 J/cm^2^
LEED	----	Low LEED: <65 J/cm Medium LEED: 65-85 J/cm High LEED: >95 J/cm
Power	0.83-6.12 W	5-30 W
Pull back speed	<1 mm/s	3-5 mm/s
Catheter/Fiber types	Concentric catheter; Eccentric catheter	Bare fiber; Radial fiber; Double-ringed radial fiber; The neverTouch fiber; Tulip fiber
Catheter/Fiber sizes	0.9-2.35 mm	350-600 μm
Energy delivery	Pulsed	Continuous; Pulsed
Target vascular types	Coronary artery; Peripheral artery	Great Saphenous Vein; Small Saphenous Vein
Indications	Acute coronary syndromes; In-stent restenosis; Noncrossable/nondilatable lesions; Chronic total occlusions; Peripheral arterial disease	Varicose veins
Ref.	[113, 118, 135, 137, 138]	[140, 142, 143, 145, 148]

HSLW: Hemoglobin-specific laser wavelengths; WSLW: Water-specific laser wavelengths; LEED: Linear endovenous energy density

## 3.Discussion

The detection of IVOCT signal depends on the optical scattering of the tissue at specific wavelength. Therefore, it can differentiate different plaque types accurately based on their optical properties. However, the maximum imaging depth of OCT is only 1.5-3 mm due to optical scattering through heterogeneous tissue, which makes it challenging to fully characterize an atheromatous plaque [67]. The IVPA can characterize the plaques by using the instinct differences in the optical absorption spectra of different tissues with ultrasound imaging depth. The development of IVPA imaging remains some challenges such as imaging speed and catheter size (< 1 mm in diameter). The rich and unique molecular-specified absorption contrast provided by PAI would be well complemented by the detailed scattering information of OCT. The multimodal PAI-OCT imaging can extract important characteristics of tissues, which has a promising future in biomedical imaging as a powerful tool for diagnostics. Widespread implementation of PAI-OCT systems depend on the development and integration of suitable light sources with high repetition rate, stable short-pulse illumination, as well as high output energy at multiple wavelengths [26]. The fiber lasers achieve a repetition rate of hundreds of kilohertz as well as pulse energy in the hundred-nanojoule range to fulfill the requirements of photoacoustic imaging applications [40, 96, 98]. The catheter size also affects imaging speed and image resolution. In order to overcome this limitation, the active size of the transducer was reduced, or the transducer was positioned obliquely [26]. It is also possible to use thinner fibers in the catheter, such as the tapered fiber, which enables small output facula, high laser energy, and obtains the high resolution, deep penetration lipids imaging [101]. With the continued advancements of new detection methods and light sources, PAI-OCT systems would be able to capture real-time large field-of-view images. Furthermore, multimodality intravascular imaging will provide high-resolution image and complementary information in microstructure and biology of plaque, as well as improving diagnostic capacity in the near future [66, 83].

Treating the specific vascular diseases through laser technologies have a better therapeutic effect. Traditional treatments are assisted by emerging laser technology to increase the success rate of treatment and reduce surgical complications. Due to the advantage of ELCA in modifying plaques, it is effective for complex coronary artery diseases. Moreover, the therapeutic effect of ELCA combined with DCB in the treatment of in-stent thrombosis is better than that of single treatment. However, laser treatment of calcified plaques is poor, the combination of ELCA and RA is effective in treating severe calcification. Careful case selection, proper use of the equipment and safe, efficacious lasing technique all play crucial roles in successful ELCA interventions. There is mounting evidence that longer-wavelength lasers targeting water might be more effective than shorter-wavelength lasers targeting haemoglobin during EVLA treatments [145]. Longer wavelengths appear to have better efficacy while minimizing side effects and allowing for lower LEED. The combination of laser imaging and therapy technology improves the success rate of laser therapy to a great extent under the guidance of laser imaging. The OCT is used to show the internal condition of blood vessels to detect the therapeutic effect of ELCA [120, 128]. Besides, real-time monitoring of the location and temperature of the fiber tip by PAI could reduce the incidence of vascular perforation and deep vein thrombosis during EVLA treatment [155, 156].

## 4.Conclusion

Laser technology has made great achievements in various fields of medicine, especially in the applications of intravascular imaging and treatment. The combination of lasers and fiber optics provides a flexible tool for imaging intravascular diseases. The emergence of multimode imaging technology can not only obtain anatomical images, but also detect vascular biochemical information. Laser technology ensures high safety in the treatment of intravascular arteriovenous diseases. Under the guidance of laser imaging, lasers can treat intravascular lesions more accurately. It can be concluded from these obvious achievements that intravascular laser imaging and therapy technologies will have a bright prospect in the field of vascular medicine.
